# Microstructure Investigation of Polymer Electrolyte Fuel Cell Catalyst Layers Containing Perfluorosulfonated Ionomer

**DOI:** 10.3390/membranes11070466

**Published:** 2021-06-24

**Authors:** Maito Koga, Hidetoshi Matsumoto, Mitsunori Kunishima, Masatoshi Tokita, Hiroyasu Masunaga, Noboru Ohta, Akihisa Takeuchi, Junji Mizukado, Hidekazu Sugimori, Kazuhiko Shinohara, Suguru Uemura, Toshihiko Yoshida, Shuichiro Hirai

**Affiliations:** 1 Department of Mechanical Engineering, Tokyo Institute of Technology, 2-12-1 Ookayama, Meguro-ku, Tokyo 152-8552, Japan; uem@eng.hokudai.ac.jp (S.U.); toshihiko_yoshida@icloud.com (T.Y.); hirai@mes.titech.ac.jp (S.H.); 2 Research Institute for Sustainable Chemistry, National Institute of Advanced Industrial Science and Technology (AIST), 1-1-1 Higashi, Tsukuba, Ibaraki 305-8565, Japan; mizukado-junji@aist.go.jp; 3 Department of Materials Science and Engineering, Tokyo Institute of Technology, 2-12-1 Ookayama, Meguro-ku, Tokyo 152-8552, Japan; mitsunori.kunishima@gmail.com; 4 Department of Chemical Science and Engineering, Tokyo Institute of Technology, 2-12-1 Ookayama, Meguro-ku, Tokyo 152-8552, Japan; tokita.m.aa@m.titech.ac.jp; 5 Japan Synchrotron Radiation Research Institute, 1-1-1 Kouto, Sayo, Hyogo 679-5198, Japan; masunaga@spring8.or.jp (H.M.); noboru_o@spring8.or.jp (N.O.); take@spring8.or.jp (A.T.); 6 Fuel Cell Cutting-Edge Research Center (FC-Cubic), Technology Research Association, AIST Tokyo Waterfront Main Building, 2-3-26 Aomi, Koto-ku, Tokyo 135-0064, Japan; h-sugimori@kri-inc.jp (H.S.); k-shinohara@fc-cubic.or.jp (K.S.); 7 School of Engineering, Hokkaido University, Kita 13, Nishi 8, Kita-ku, Sapporo, Hokkaido 060-8628, Japan

**Keywords:** polymer electrolyte fuel cell, ionomer, catalyst layer, transmission electron microscopy, synchrotron X-ray scattering

## Abstract

Perfluorosulfonated ionomers are the most successful ion-exchange membranes at an industrial scale. One recent, cutting-edge application of perfluorosulfonated ionomers is in polymer electrolyte fuel cells (PEFCs). In PEFCs, the ionomers are used as a component of the catalyst layer (CL) in addition to functioning as a proton-exchange membrane. In this study, the microstructures in the CLs of PEFCs were characterized by combined synchrotron X-ray scattering and transmission electron microscopy (TEM) analyses. The CL comprised a catalyst, a support, and an ionomer. Fractal dimensional analysis of the combined ultrasmall- and small-angle X-ray scattering profiles indicated that the carbon-black-supported Pt catalyst (Pt/CB) surface was covered with the ionomer in the CL. Anomalous X-ray scattering revealed that the Pt catalyst nanoparticles on the carbon surfaces were aggregated in the CLs. These findings are consistent with the ionomer/catalyst microstructures and ionomer coverage on the Pt/CB surface obtained from TEM observations.

## 1. Introduction

**Figure 1:**
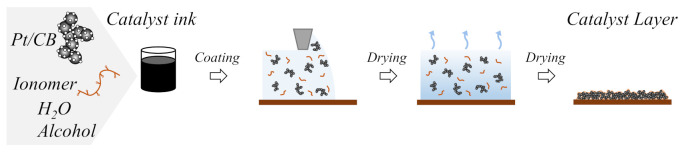
Fabrication of the catalyst layer from catalyst ink.

Perfluorosulfonated ionomers are the most successful ion-exchange membranes at an industrial scale [1]. The ionomers are commonly used as the proton-exchange membrane in polymer electrolyte fuel cells (PEFCs) [2]. Recently, PEFCs have become the most attractive type of electrochemical power converter thanks to their wide variety of applications, such as in automotive power, stationary power, and microelectronics [3,4]. PEFCs convert the chemical energy of hydrogen and oxygen fuels directly into electricity, affording devices with high power density, zero CO_2_ emissions, and low operating temperatures. In PEFCs, the ionomers are used as components of the catalyst layers (CL) in addition to being used as the proton-exchange membrane. The CL in PEFCs is fabricated by catalyst ink coating (Figure 1) and plays a vital role in determining the PEFC’s performance [5]. Ionomers in the CL that function as dispersion agents and binders cover the carbon-supported Pt catalysts and provide the proton transport pathways necessary for electrochemical reactions (i.e., oxygen reduction and hydrogen oxidation) [6]. Ionomers also significantly influence the permeation of reactant gases to catalyst sites, Pt activity, the transport of water to and from reaction sites, and the formation of an interconnected carbon and pore structure in CLs [4,5,6,7]. Therefore, an in-depth understanding of CL microstructures is required for their rational design in order to fabricate PEFCs with improved performance.

The microstructures of the catalyst inks and CLs have been extensively studied both theoretically [8,9] and experimentally [10,11,12,13,14,15,16,17,18,19,20,21] using techniques such as conventional and cryogenic scanning and transmission electron microscopy (SEM and TEM) [10,11,12,13,14,15,16], focused ion beam SEM (FIB-SEM) [17,18], atomic force microscopy [10,19], X-ray computed tomography [20], and X-ray [13,16] and neutron scattering [21]. Among the scattering techniques, anomalous small-angle X-ray scattering (ASAXS) is a powerful technique that can provide element-specific data from a multicomponent system [22,23,24,25] and has been used to observe the size distribution of Pt nanoparticles (NPs) in carbon-supported Pt catalysts [22,23,24]. However, studies carrying out systematic structural analysis of the CLs with hierarchical and inhomogeneous structure are limited.

In this study, we investigated the microstructures of ionomers and carbon-black-supported Pt catalysts (Pt/CB) in CLs by combining synchrotron X-ray scattering techniques and TEM observations. We focused on the adsorption of the ionomer on Pt/CB and the formation of Pt aggregates in CLs. Combined USAXS and SAXS as well as ASAXS analyses were conducted to obtain information on the multiscale CL structures and the Pt catalyst’s structure over a large sample area, respectively. Additionally, the combination of X-ray scattering and TEM enabled the accurate analysis of the complex scattering profiles of the CLs.

## 2. Materials and Methods

### 2.1. Materials

A 20 wt% Nafion^®^ dispersion (DE2020 CS type: 34 wt% water, 44 wt% 1-propanol (NPA), and 2 wt% other volatile organic compounds) was purchased from Chemours (Wilmington, USA). Ethanol (EtOH, 99.5%) and *N*, *N*-dimethylformamide (DMF, 99.0%) were purchased from Fujifilm Wako Pure Chemical Corporation (Osaka, Japan). The Pt/CB (TEC10V30E, Pt loading: 30 wt%) was purchased from Tanaka Kikinzoku Kogyo (Tokyo, Japan). Reagents were used without further purification. Ultra-pure water produced using a Milli-Q water purification system (Direct-Q 3UV, Millipore, Bedford, MA, USA) was used for all measurements.

### 2.2. Catalyst Ink Preparation

The catalyst ink was prepared from catalyst powder (Pt/CB), ionomer (Nafion) dispersion, and solvents (EtOH and H_2_O) as per the composition listed in Table 1. The samples were prepared by dispersing a Pt/CB in H_2_O using a planetary centrifugal mixer (Awatori-rentaro, ARE-301, THINKY, Ltd., Tokyo, Japan). After 1 min of mixing the Pt/CB dispersion, the required volume of the ionomer dispersion was added. After mixing the resulting dispersion for 1 min, EtOH was added. The catalyst ink contained 9.5 wt% solids, of which the ionomer comprised 3.3 wt%. The ionomer to carbon support weight ratio (I/C) was 0.75. After 1 min of mixing, the final dispersion was mixed using a high-speed rotary-type mixer (Filmix 30-L, PRIMIX Corporation, Awaji, Japan) at 22,000 rpm for 10 min, after which the catalyst ink was obtained. For comparison, a 0.2 wt% Pt/CB dispersion in DMF without the ionomer was prepared by ultrasonication.

The catalyst ink was coated on a polyimide film fixed on a silicon (Si) substrate using an automatic film applicator (No. 605S, Mys-Tester Company, Ltd., Ikeda, Japan) equipped with a 40-mm-wide stainless-steel (SUS316L) blade at a gap of 100 μm at 20 mm s^−1^ at 25 ± 0.5 °C.

### 2.3. TEM Observations

Pt/CB powder was dispersed in water at a concentration of 10^-4^ wt% and deposited on the lacey carbon films on copper mesh TEM grids. The CLs were held between the epoxy resin blocks and sectioned to a thickness of ~200 nm using an ultramicrotome (Leica UC6/FC6, Leica Microsystems GmbH, Wetzlar, Germany) at −80 °C. The ultrathin sections were transferred to lacey carbon films on copper mesh TEM grids. TEM observations were carried out on a JEM-ARM300F (JEOL Ltd., Akishima, Japan) instrument operating at 300 kV and equipped with a CMOS camera (OneView, Gatan Inc., Pleasanton, CA, USA).

### 2.4. X-Ray Scattering

USAXS, SAXS, and ASAXS measurements were performed at SPring-8, Hyogo, Japan. SAXS measurements were conducted at the BL40B2 beamline: the CL coated on a polyimide film was irradiated with X-rays of wavelength (*λ*) = 0.1 nm. The scattering patterns were recorded on a PILATUS3 S 2M detector (Dectris, Baden-Daettwil, Switzerland) located 4 m from the sample. USAXS measurements were performed at the BL03XU and BL20XU beamlines. The CLs were irradiated with X-rays of λ = 0.2 and 0.0539 nm. Scattering patterns were recorded on PILATUS3 S 1M and PILATUS 300 K detectors (Dectris) located 8 m and 160 m from the samples, respectively. The SAXS intensity profiles were prepared by circularly averaging the intensity on 2D images and plotting the intensity against the magnitude of scattering vector *q* = 4π sin *θ*/*λ*, where 2*θ* is the scattering angle. Although CLs display SAXS patterns originating from structures formed by Pt and CB, the scattering coming from Pt can be extracted using the ASAXS method. The SAXS intensity depends on the atomic form factors, *f* (*q*, *E*), of each element, where *E* is the incident X-ray energy. With decreasing *E* (i.e., increasing λ), the magnitude of *f* (*q*, *E*) drops on crossing the absorption edge. Pt has a Pt-L_3_ absorption edge at *E* = 11.564 keV. Thus, the intensity of SAXS ascribed to structures of Pt, *I*_Pt_ (*q*), is calculated from three SAXS data measured using X-rays with different wavelengths near 0.1 nm, namely, *I* (*q*, *E*_k_) with *E*_1_ = 11.550 keV (*λ*_1_ = 0.10736 nm), E_2_ = 11.560 keV (*λ*_2_ = 0.10727 nm), and *E*_3_ = 11.562 keV (*λ*_3_ = 0.10725 nm). The measurements were conducted at the BL40B2 beamline. The scattering patterns were recorded on a PILATUS3 S 2M detector located 2 m from the sample. *I*_Pt_ (*q*, *E*) was calculated using the following equation [24]:

(1) $$I_{\mathrm{Pt}}\left(q,\;E\right)\;\sim \;\frac{I\left(q,\;E_{1}\right)-I\left(q,\;E_{2}\right)}{f^{\prime }\left(q,E_{1}\right)-f^{\prime }\left(q,E_{2}\right)}-\frac{I\left(q,\;E_{1}\right)-I\left(q,\;E_{3}\right)}{f^{\prime }\left(q,E_{1}\right)-f^{\prime }\left(q,E_{3}\right)}$$

where *f*′ is the real part of the complex atomic form factor of Pt, and the values used were *f*′ (*E*_1_) = 54.03, *f*′ (*E*_2_) = 52.71, and *f*′ (*E*_3_) = 52.45 electrons.

## 3. Results and Discussion

**Figure 2:**
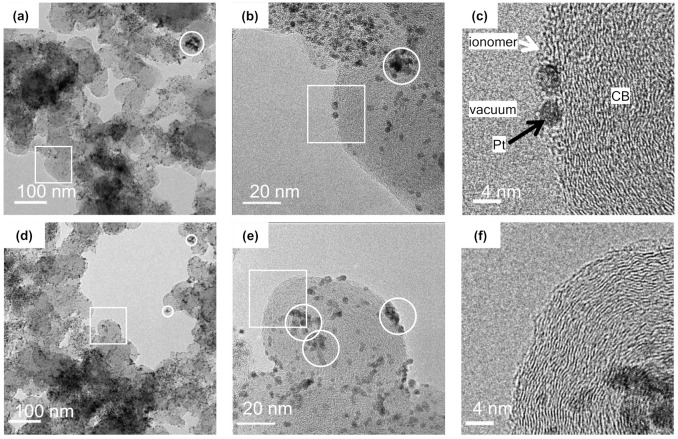
Typical TEM images of the CL (**a**–**c**) and Pt/CB (**d**–**f**). (**b**) and (**e**) are magnified images of the square in (**a**) and (**d**), respectively; and (**c**) and (**f**) are the magnified images of the square in (**b**) and (**e**), respectively. The circle shows the aggregates of Pt nanoparticles.

The CL TEM micrographs (Figure 2a–c) show that the ionomers cover the Pt/CB surface. The adsorbed ionomers form a layered structure with a thickness of several nanometers on the surface of the CB and Pt NPs (Figure 2c); the Pt NPs aggregates are circled. Figure 2 d–f show the Pt/CB powder TEM micrographs for comparison. The bright region corresponds to a vacuum; the light gray and dark gray/black particles correspond to the CB aggregates and Pt NPs on the CB, respectively (Figure 2d). The CB aggregates appear to be larger than 100 nm and are composed of CB particles of size 20–40 nm, while the Pt NPs are ~3 nm in size (Figure 2e). The CB nanostructure originates from the concentric stacking of graphite-like carbon layers, with an interlayer distance of ~0.35 nm and a lateral extension of several nanometers (Figure 2f). This carbon layer stacking locally generates crystal defects, resulting in the formation of an uneven structure on the CB surface. Upon comparing the Pt/CB surface with (Figure 2a–c) and without (Figure 2d–f) ionomer, the Pt/CB surface TEM micrograph appears smoother due to the ionomers present on the Pt/CB agglomerates (supporting TEM images are shown in Figure S1, Supplementary Material).

**Figure 3:**
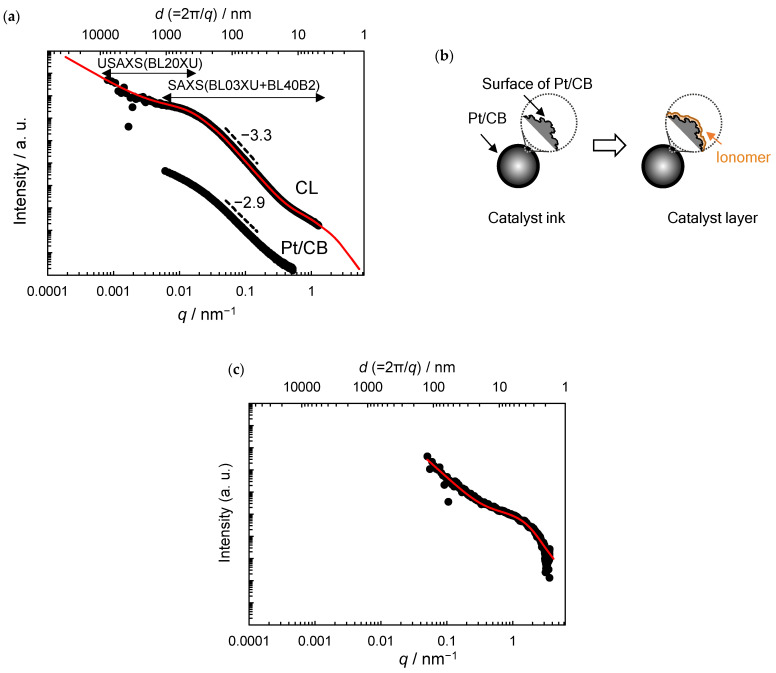
(**a**) The combined USAXS and SAXS intensity profiles of the CL and Pt/CB. The red solid lines in (**a**) are the fitting curve obtained using Equation (2). (**b**) Plausible model of Pt/CB surface structure. (**c**) ASAXS intensity profile of the CL calculated using Equation (1). The red solid lines in (**c**) are the fitting curve obtained using Equation (3).

Figure 3a shows the combined CL USAXS and SAXS intensity profiles. The deflections appear at *q* ≈ 0.02 nm^−1^ and 0.3 nm^−1^. These deflections indicate that the CL has at least two structures with different sizes (i.e., radii of gyration of *R*_g1_ and *R*_g2_). For the combined profile analysis, the Beaucage unified equation, commonly used to investigate the hierarchical structure of inhomogeneous polymer/CB nanocomposites, was adopted [26,27,28,29]. The optimized unified equation for the CL is:

(2) $$\begin{array}{l} I\left(q\right)=Aexp{\left(-q^{2}R_{g1}{}^{2}/3\right)}^{-D_{m1}}+Bexp\left(-q^{2}R_{g1}{}^{2}/3\right)\\ \;\;\;\;\;+Cexp\left(-q^{2}R_{g2}{}^{2}/3\right)\times {\left[{\left(erf\frac{qR_{g1}}{\sqrt{6}}\right)}^{3}/q\right]}^{\left(6-D_{s1}\right)}\\ \;\;\;\;\;+Dexp\left(-q^{2}R_{g2}{}^{2}/3\right)+E{\left[{\left(erf\frac{qR_{g2}}{\sqrt{6}}\right)}^{3}/q\right]}^{\left(6-D_{s2}\right)} \end{array}$$

where *R*_g1_ and *R*_g2_ are the radii of gyration of the two structures, respectively, *D*_m1_ is the mass fractal dimension, *D*_s1_ and *D*_s2_ are the surface fractal dimensions of the two structures, and *A*, *B*, *C*, *D*, and *E* are proportional constants.

The curve (solid red line) calculated using Equation (2) agrees well with the experimental results (black dots). The best-fitting values are *R*_g1_ = 100 nm, *R*_g2_ = 1.7 nm, *D*_m1_ = 1.7, *D*_s1_ = 2.7, and *D*_s2_ = 2.0. The estimated radii of the sphere (*R* = (3/5) − 1/2*R*_g_) for the two structures are 129 nm and 2.2 nm, which correspond to the size of the CB aggregates and Pt NPs, respectively (Figure 2d–f). Note that the USAXS data are limited in the low *q* region, but the profile at *q* < 0.005 nm^−1^ suggests the formation of the mass fractal objects formed by CB aggregates with *D*_m1_ of 1.7 (denoted CB agglomerates) [27,28].

The SAXS intensity profile of the Pt/CB dispersion in DMF is shown in Figure 3a for comparison. At 0.05 < *q* < 0.15 nm^−1^, the power-scattering profile is given by *I* (*q*)~*q*^−2.9^ and the *D*_s_ for Pt/CB without ionomer is estimated to be 3.1. The difference in *D*_s_ between the CL (2.7) and Pt/CB (3.1) clearly indicates the formation of a smoother Pt/CB surface due to the adsorption of ionomer, consistent with the ionomer coverage of the Pt/CB surface in the CLs, observed in the TEM images (Figure 2b,c,e,f). The plausible surface structure of Pt/CB is shown in Figure 3b.

The CL was also analyzed using ASAXS to investigate the contribution of Pt to the scattering intensity profiles. Figure 3c shows the CL *I*_Pt_ (*q*) profiles calculated using Equation (1). A deflection appears at *q* ≈ 0.3 nm^−1^, indicating a single structure with *R*_g2_. Therefore, the following Beaucage unified equation was adopted [27]:

(3) $$\begin{array}{l} I\left(q\right)=Cexp{\left(-q^{2}R_{g2}{}^{2}/3\right)}^{-D_{m2}}+Dexp\left(-q^{2}R_{g2}{}^{2}/3\right)\\ \;\;\;\;\;\;+E{\left[{\left(erf\frac{qR_{g2}}{\sqrt{6}}\right)}^{3}/q\right]}^{\left(6-D_{s2}\right)} \end{array}$$

The curve (solid red line) calculated using Equation (3) using radius *R*_g2_ agreed well with the experimental results (black dots). The best-fitting values were *R*_g2_ = 1.3, *D*_m2_ = 2.7, and *D*_s2_ = 2.0. The estimated radius of sphere *R* is 1.7 nm, which compares more favorably with the radius of Pt NPs (1.5 nm) determined by TEM observations (Figure 2b,c) than that obtained by SAXS analysis using Equation (2) (2.2 nm) containing the contribution of the coated Nafion. Note that the *q*-range in the ASAXS data is smaller compared with that in the combined SAXS and USAXS data; however, the profile at *q* < 0.1 nm^−1^ clearly indicates the formation of the mass fractal aggregates of Pt NPs with a *D*_m2_ of 2.7, consistent with the Pt NPs aggregates in the CL (originally from the Pt/CB powder), observed in the TEM images (the circles in Figure 2).

## 4. Conclusions

We investigated the ionomer/catalyst microstructures in the CLs of PEFCs using synchrotron X-ray scattering techniques and TEM. The ionomer adsorption on the Pt/CB surface in the CLs was analyzed based on the combined USAXS and SAXS profiles. The fractal dimensional analysis of the scattering profiles clearly indicated that the Pt/CB surface was covered with ionomer in the CL. ASAXS analyses demonstrated that the Pt nanoparticle aggregates formed in the CLs. These findings are consistent with the ionomer/catalyst microstructures and ionomer coverage of the Pt/CB surface in the CLs characterized by TEM observations. The combination of scattering techniques and microscopic structural analysis enabled a systematical exploration of the structural information of multicomponent and multiscale systems. The insights provided by this approach will be useful for the rational microstructural design of CLs and for monitoring the stability of CLs (e.g., detected by microstructural change). Our future work would include the relationship between the CL microstructure and the MEA performance and/or durability of the CLs. In addition, this approach can be applied to polyelectrolyte nanocomposites [30] and porous electrodes [31] comprising an active material and a polymer, used in various energy conversion and storage devices, including FCs and other batteries.

## Figures and Tables

**Table 1:** Composition of catalyst ink.

Component	Pt	CB	Nafion	H_2_O	NPA	EtOH
Content (wt%)	1.8	4.4	3.3	50.6	5.6	34.4

## Supplementary Materials

The following are available online at https://www.mdpi.com/article/10.3390/membranes11070466/s1, Figure S1: Additional TEM images of the CL.
