# Supplementary material for: Microstructure Investigation of Polymer Electrolyte Fuel Cell Catalyst Layers Containing Perfluorosulfonated Ionomer
Source: Membranes (Basel). 2021 Jun 24;11(7):466. doi: 10.3390/membranes11070466 (PMC8307432; doi:10.3390/membranes11070466)
Supplement: Supplementary file 1 [file membranes-11-00466-s001.zip › membranes-1246462-supplementary.pdf]

Supplementary Materials:

Microstructure Investigation of Polymer Electrolyte Fuel Cell Catalyst Layers Containing Perfluorosulfonated Ionomer

Maito Koga, Hidetoshi Matsumoto, Mitsunori Kunishima, Masatoshi Tokita, Hiroyasu Masunaga, Noboru Ohta, Akihisa Takeuchi, Junji Mizukado, Hidekazu Sugimori, Kazuhiko Shinohara, Suguru Uemura, Toshihiko Yoshida, and Shuichiro Hirai

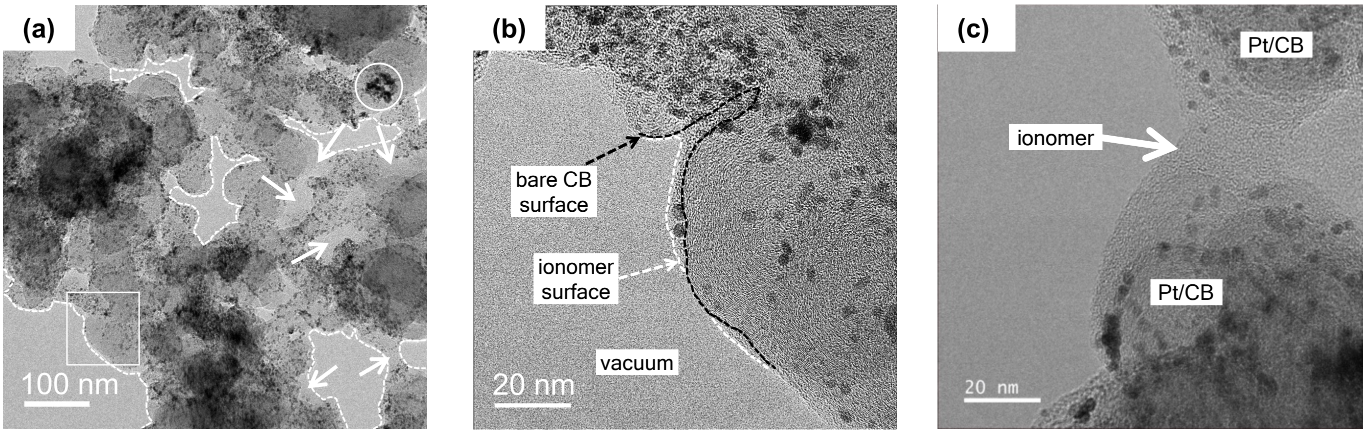

Figure S1 Additional TEM images of the CL. The original images of (**a**) and (**b**) are shown in Figure 2. (**b**) is magnified image of the square in (**a**). The white dotted lines in (**a**,**b**) show the ionomer surface and the black dotted lines in (**b**) show the CB surface. The white solid arrows in (**a**,**c**) point to the region filled with ionomer. In the CLs, the spaces between Pt/CB agglomerates are filled with ionomer.
